# Multiple Adenylate-Forming Enzymes Contribute to Biosynthesis of the DPO Quorum-Sensing Autoinducer

**DOI:** 10.1021/acschembio.5c00932

**Published:** 2026-01-30

**Authors:** Delaney M. Lacey, Gabriel D. D’Agostino, Emilee E. Shine, Bonnie L. Bassler

**Affiliations:** † Department of Molecular Biology, 6740 Princeton University, Princeton, New Jersey 08544, United States; ‡ Howard Hughes Medical Institute, Chevy Chase, Maryland 20815, United States

## Abstract

Bacteria use a process of chemical communication called quorum sensing to regulate group behaviors. Quorum sensing relies on the synthesis, release, and detection of signal molecules called autoinducers that accumulate with increasing cell density. The pathogen *Vibrio cholerae* makes and detects three autoinducers which together, regulate genes required for group behaviors including virulence and biofilm formation. Two autoinducers are produced by dedicated autoinducer synthases that employ *S*-adenosyl methionine as a substrate. The third autoinducer, 3,5-dimethylpyrazin-2-ol (DPO), is produced from threonine and alanine. The threonine dehydrogenase (Tdh) enzyme oxidizes l-threonine to 2-amino-3-ketobutyric acid, which spontaneously decarboxylates to aminoacetone. Here, we define the steps required to convert aminoacetone and alanine into DPO. We show that diverse adenylate-forming enzymes can condense ATP and d- or l-alanine to form alanyl-adenylate, the necessary intermediate in DPO biosynthesis. Upon release, alanyl-adenylate spontaneously condenses with aminoacetone to form *N*-alanyl-aminoacetone, which cyclizes to form DPO. We propose that DPO is distinct from other autoinducers in that there is apparently no dedicated synthase. Rather, a collection of enzymes contribute to the production of this quorum-sensing autoinducer.

## Graphical abstract

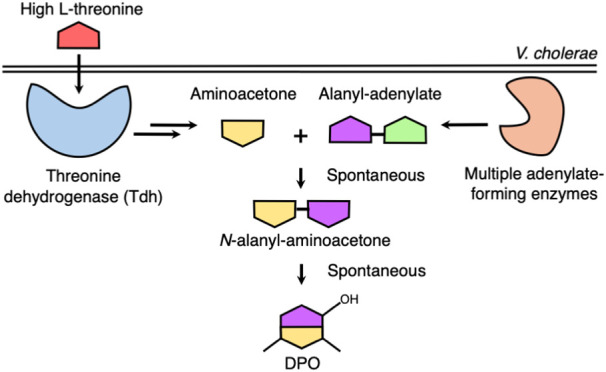

## Introduction

Quorum sensing (QS) is a process of cell–cell communication that bacteria use to orchestrate collective behaviors. QS bacteria synthesize, release, and detect extracellular signal molecules called autoinducers that accumulate in step with increasing cell density.^1^ In Gram-negative bacteria, most autoinducers, irrespective of their varied structures, are made by dedicated autoinducer synthases that rely on *S*-adenosyl methionine (SAM) as a substrate.^2^ In vibrios, in addition to two SAM-dependent autoinducers called CAI-1 and AI-2, there is a third autoinducer, 3,5-dimethyl-pyrazin-2-ol (DPO).^3–5^ DPO is detected by the VqmA receptor, a transcription factor. Upon binding DPO, VqmA activates the expression of *vqmR*, encoding the VqmR regulatory small RNA. At high cell density, VqmR suppresses genes required for virulence factor production and biofilm formation in *Vibrio cholerae*, the bacterium in which the DPO-VqmA-VqmR system has been best characterized. This activity allows *V. cholerae* to disperse from the human host.^5,6^ DPO is broadly made by microorganisms beyond vibrios, both bacterial and eukaryotic, suggesting the possibility that DPO fosters communication across species and possibly across domains.^7–9^

Most of the DPO biosynthetic pathway is undefined and, hence, the topic of the present work. What is known is that DPO is a member of a class of pyrazinone molecules that rely on the threonine dehydrogenase (Tdh) enzyme for their production. Indeed, in all DPO-producing bacterial species tested, when the *tdh* gene is eliminated, the bacteria become incapable of DPO production.^5,10^ Tdh oxidizes l-threonine to 2-amino-3-ketobutyric acid, which spontaneously decarboxylates to aminoacetone (Figure 1). Based on the known DPO structure, the steps to convert aminoacetone to DPO are presumed to require an enzyme-catalyzed condensation reaction that involves alanine. Consistent with this model, amino acid labeling studies show that alanine and threonine are both incorporated into DPO.^5^ Transposon mutagenesis screens to identify genes required for DPO production revealed *tdh*, but no other genes.^5,10^ These results suggested that if other enzymes are required for DPO biosynthesis, they are either essential or redundant.

**Figure 1:**
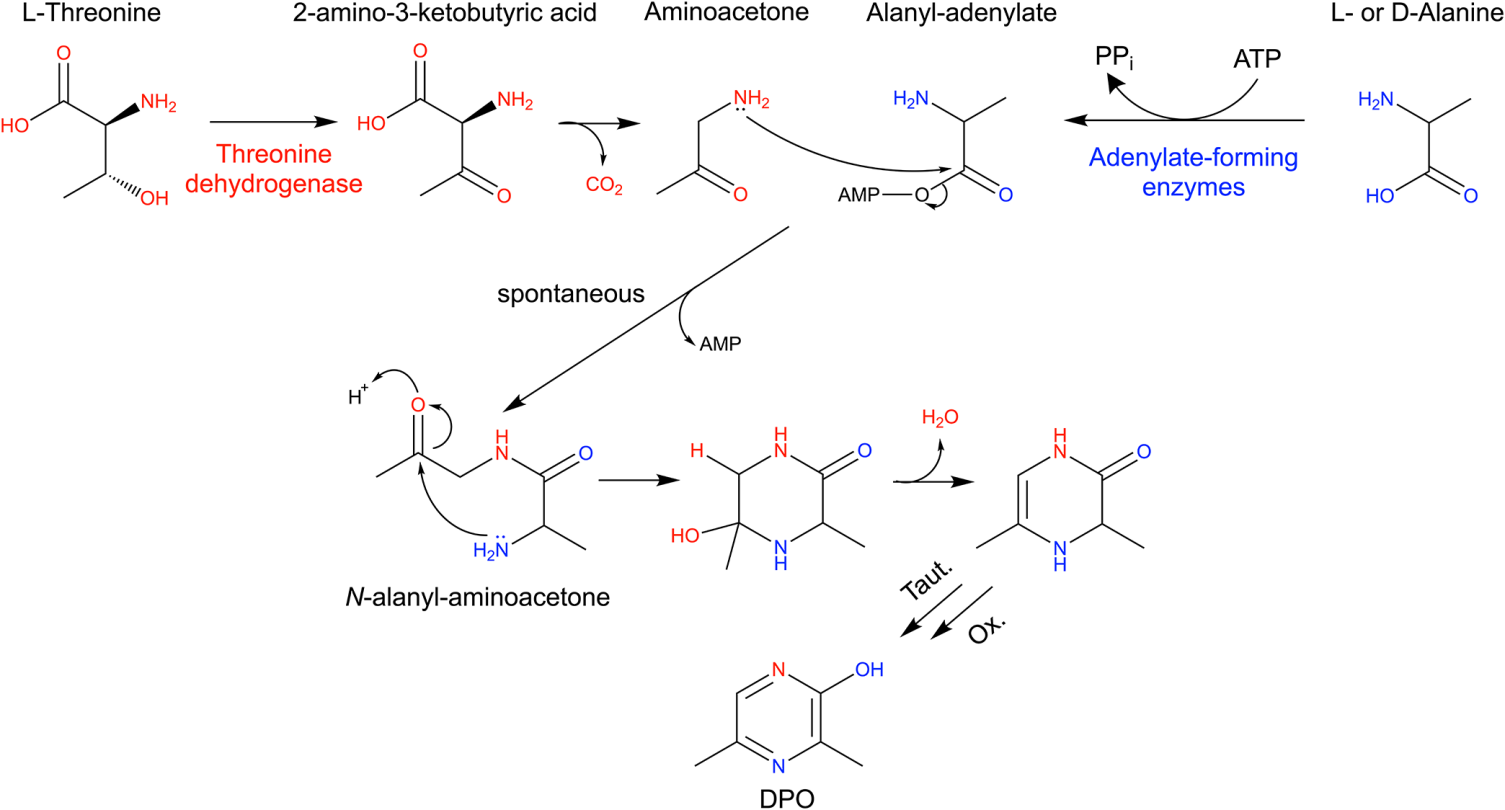
Adenylate-forming enzymes contribute to biosynthesis of the DPO autoinducer. DPO is produced from threonine and alanine. The threonine dehydrogenase (Tdh) enzyme converts l-threonine to 2-amino-3-ketobutyric acid, which spontaneously decarboxylates to aminoacetone. Multiple-adenylate forming enzymes bind d- or l-alanine and ATP to form alanyl-adenylate. Upon release, alanyl-adenylate spontaneously condenses with aminoacetone to form *N*-alanyl-aminoacetone, which cyclizes to form DPO.

Pyrazinone molecules similar to DPO have been identified in *Escherichia coli*. Analogous to DPO, production of these other pyrazinones relies on l-threonine and Tdh. However, unlike DPO, amino acids other than alanine are employed in their biosynthesis. Aminoacyl-tRNA synthetases, the enzymes responsible for charging tRNAs with amino acids, are suggested to promote the condensation of aminoacetone with the corresponding amino acids to make the linear precursors to these pyrazinones.^10^ However, the putative linear precursor to DPO, *N*-alanyl-aminoacetone, has never been detected *in vivo*, likely due to its high reactivity.^10,11^ Using synthetic compounds, we previously showed that both DPO and *N*-alanyl-aminoacetone bind to and activate VqmA.^11^

Here, we demonstrate that aminoacetone, produced by Tdh, and alanyl-adenylate, derived from l- or d-alanine and ATP, spontaneously condense to form *N*-alanyl-aminoacetone, which cyclizes to produce DPO (Figure 1). Importantly, we show that multiple adenylate-forming enzymes can contribute to the production of the required alanyl-adenylate intermediate that reacts with aminoacetone to produce DPO. Specifically, we tested 5 such enzymes and demonstrate they make *N*-alanyl-aminoacetone that can cyclize to DPO. These enzymes include several aminoacyl-tRNA synthases, including ones from different structural classes and which activate different amino acids as well as carrier protein acyl-ligases. We show that proclivity for alanyl-adenylate production, rather than conserved structural features, is the key predictor of whether an adenylate-forming enzyme will readily contribute to DPO production. *N*-alanyl-aminoacetone production by adenylate-forming enzymes increases linearly with time, suggesting that once alanyl-adenylate is enzymatically produced and released, it reacts spontaneously with aminoacetone to generate *N*-alanyl-aminoacetone, which cyclizes to form DPO. Overexpression of vibrio *almE*, the gene encoding one of the above adenylate-forming enzymes, in *E. coli* increased DPO activity. However, deletion of *almE* from its native *V. cholerae* host did not affect levels of DPO activity. Together, these findings suggest that, *in vivo*, a collection of enzymes contribute to global DPO production. Thus, DPO is distinct from other autoinducers in that there is apparently no dedicated synthase. Rather, a suite of enzymes must divert resources from their established functions to contribute to the production of this QS autoinducer.

## Results

### Ala-AMP is the Intermediate and *N*-alanyl-aminoacetone is the Precursor to the DPO Autoinducer

The DPO autoinducer is made from threonine and alanine. The Tdh enzyme converts l-threonine to 2-amino-3-ketobutyric acid, which spontaneously decarboxylates to aminoacetone. Aminoacetone and alanine cannot spontaneously generate DPO.^5^ Thus, an unidentified enzyme must activate alanine enabling it to condense and cyclize with aminoacetone to produce DPO. Aminoacyl-tRNA synthetases have been shown to produce linear precursors to pyrazinones *in vitro* and thus, we reason they may similarly facilitate production of DPO or its proposed linear precursor *N*-alanyl-aminoacetone.^10,5^

Canonically, a tRNA-synthetase charges a cognate tRNA with a partner amino acid in a two-step reaction. In the first step, the amino acid is activated by ATP in the tRNA-synthetase catalytic site, forming an aminoacyl-adenylate moiety and releasing pyrophosphate (PP_i_). In the second step, the amino acid is transferred to the tRNA, and AMP is released.^12^ Given the connection between tRNA-synthetases and pyrazinones, we wondered if alanyl-tRNA synthetase (AlaRS), the synthetase specific for l-alanine (l-Ala), could produce DPO. To test this possibility, reactions containing purified *E. coli* AlaRS, ATP, aminoacetone, ^13^C_3_-l-Ala, and inorganic pyrophosphatase were carried out. Following reaction termination, the samples were applied to a Δ*tdh V. cholerae* strain harboring a *vqmR-lux* transcriptional reporter construct.^6,13^ The logic is as follows: VqmA binds DPO and the linear *N*-alanyl-aminoacetone precursor. The VqmA-DPO and VqmA-*N*-alanyl-aminoacetone complexes activate expression of *vqmR*.^5,11^ Therefore, light production from the *vqmR-lux* fusion can be used to assess the presence, absence, and amounts of active molecule, with concentrations approximated by comparison to *vqmR-lux* reporter activity generated from known amounts of synthetic ^13^C-DPO or synthetic *d*_3_-*N*-alanyl-aminoacetone. *d*_3_-*N*-alanyl-aminoacetone is as potent as ^13^C-DPO in this assay (Figure S1A). Incorporation of the *vqmR-lux* fusion into a Δ*tdh V. cholerae* strain ensures that reporter output occurs exclusively in response to exogenously supplied compounds. The strain reports only on signal transduction through the VqmA-directed QS pathway.^5,11,13^ From here forward, we call the bioluminescence assay employing Δ*tdh V. cholerae* carrying *vqmR-lux* “the Lux bioassay”.

In parallel with the Lux bioassay, and from the same reaction mixtures, we quantified ^13^C_3_-alanyl-adenylate (^13^C_3_-Ala-AMP) production by AlaRS. Because Ala-AMP is highly labile, direct measurement is not possible, thus, as a surrogate, we measured pyrophosphate (PP_i_), the obligatory 1:1 product of AlaRS-catalyzed ^13^C_3_-Ala-AMP formation.^14^ We used inorganic pyrophosphatase to convert PP_i_ to 2P_i_, which was measured using a spectrophotometric malachite green technique.^15,16^ We verified that the pyrophosphatase completely hydrolyzed PP_i_ to 2P_i_ under our assay conditions. PP_i_ production was quantified using standard curves generated with known concentrations of P_i,_ where [P_i_]/2 equals [PP_i_] produced and thus, [^13^C_3_-Ala-AMP] generated (Figure S1B). Reactions were normalized to samples lacking the AlaRS enzyme.

Figure 2A shows that reaction mixtures containing all the reactants yielded activity in the Lux bioassay whereas there was no activity when AlaRS, ATP, aminoacetone, or ^13^C_3_-l-Ala was omitted. Likewise, if BSA was substituted for AlaRS, no activity was produced. Addition of pyrophosphatase increased activity by ∼50-fold. Pyrophosphatase is known to enhance tRNA synthetase-driven aminoacyl-adenylate formation because it hydrolyzes the feedback inhibitor PP_i_, to 2P_i_.^17,18^ For this reason, and to measure ^13^C_3_-Ala-AMP formation (Figures 2B and S1B), unless otherwise indicated, pyrophosphatase is included in all reactions reported here.

**Figure 2:**
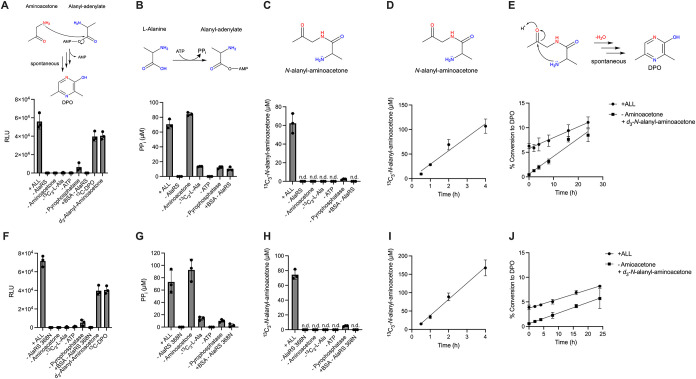
Ala-AMP is the intermediate and *N*-alanyl-aminoacetone is the DPO precursor. *In vitro* reactions were carried out with 1 μM WT AlaRS enzyme (panels A-E) or the aminoacylation defective enzyme AlaRS 368N (panels F-J), ATP [2 mM], aminoacetone [1 mM], ^13^C_3_-l-Ala [1 mM], and inorganic pyrophosphatase [0.5 U/mL]. Reactions were terminated after 2 h (panels A-C and F–H). (A, F) Quantitation of activity from *in vitro* reactions using the Lux bioassay. RLU denotes relative light units. Reactions were normalized to samples lacking enzyme. (B, G) Quantitation of PP_i_ production from *in vitro* reactions using the malachite green assay. PP_i_ was quantified using standard curves generated with known concentrations of P_i_ where [P_i_]/2 equals [PP_i_] (see Figure S1B). Since PP_i_ and ^13^C_3_-Ala-AMP are the adenylation reaction products and are made at a 1:1 ratio, measurement of [PP_i_] is used as a proxy for [^13^C_3_-Ala-AMP]. Reactions were normalized to samples lacking enzyme. (C, H) Quantitation of ^13^C_3_-*N*-alanyl-aminoacetone from *in vitro* reactions assessed by UPLC-MS. ^13^C_3_-*N*-alanyl-aminoacetone concentration was determined using standard curves generated from known concentrations of synthetic *d*_3_-*N*-alanyl-aminoacetone (see Figure S1C). (D, I) Quantitation of the ^13^C_3_-*N*-alanyl-aminoacetone produced over time in the *in vitro* reactions assessed by UPLC-MS. (E, J) Percent conversion of ^13^C_3_-*N*-alanyl-amioacetone produced by the *in vitro* enzymatic reactions to ^13^C_3_-DPO or of synthetic *d*_3_-*N*-alanyl-aminoacetone to *d*_3_-DPO following heating to 37 °C degrees assessed by UPLC-MS. DPO yield increases with increasing concentration of the supplied *N*-alanyl-aminoacetone (see Figure S1G). Linear regression analyses were used to fit the data (solid lines); the R-squared values are 0.9252 and 0.9676 for AlaRS and AlaRS 368N (D and I, respectively), 0.8118 and 0.9645 (+ALL, E and J respectively), and 0.9330 and 0.8648 (-Aminoacetone, + *d*_3_-*N*-alanyl-aminoacetone, E and J respectively). In all panels, error bars denote standard deviations of three biological replicates. The reaction step or product measured is shown above each panel. Red and blue heteroatoms denote, respectively, the atoms contributed by threonine and alanine to DPO.

To confirm that the Lux bioassay activity can be attributed to ^13^C_3_-DPO or ^13^C_3_-*N*-alanyl-aminoacetone production, we analyzed the reaction mixtures by ultra-performance liquid chromatography mass spectrometry (UPLC-MS). ^13^C_3_-*N*-alanyl-aminoacetone (^13^C_3_C_3_H_12_N_2_O_2_, [M + H]+: 148.1) and ^13^C_3_-DPO (^13^C_3_C_3_H_8_N_2_O, [M + H]+: 128.1) were only detected by UPLC-MS in samples with AlaRS, ATP, aminoacetone, and ^13^C_3_-l-Ala. As expected, the addition of pyrophosphatase increased ^13^C_3_-*N*-alanyl-aminoacetone production. To quantify ^13^C_3_-*N*-alanyl-aminoacetone and ^13^C_3_-DPO in the UPLC-MS analyses, we used standard curves generated from known concentrations of synthetic *d*_3_-*N*-alanyl-aminoacetone and ^13^C-DPO (Figure S1C,D). Our reaction conditions resulted in a ∼6% yield of ^13^C_3_-*N*-alanyl-aminoacetone (Figure 2C). Below, we deduce the reaction mechanism and show why a modest yield should be expected at our 2 h assay time point (Figure 2D). ^13^C_3_-DPO was detected, and its concentration increased when the samples were heated to 37 °C after reaction termination. Approximately 10% of the AlaRS-derived ^13^C_3_-*N*-alanyl-aminoacetone cyclized to ^13^C_3_-DPO under those conditions. Importantly, a similar level of conversion of *d*_3_ *-N*-alanyl-aminoacetone to *d*_3_ *-*DPO occurred when synthetic *d*_3_ *-N*-alanyl-aminoacetone was incubated at 37 °C with all reaction components except aminoacetone (Figure 2E).

^13^C_3_-*N*-alanyl-aminoacetone production in the above reactions accounts for the activity in the Lux bioassay (Figure S1E–F). Based on measurements of ^13^C_3_-*N*-alanyl-aminoacetone (Figure 2C) and PP_i_ and thus,^13^C_3_-Ala-AMP produced (Figure 2B), there appears to be a 1:1 conversion of ^13^C_3_-Ala-AMP to ^13^C_3_-*N*-alanyl-aminoacetone. These data suggest that AlaRS uses ATP and ^13^C_3_-l-Ala to generate ^13^C_3_-Ala-AMP, which condenses with aminoacetone to form ^13^C_3_-*N*-alanyl-aminoacetone, which in turn can spontaneously cyclize over time to form ^13^C_3_-DPO (Figure 1). However, like other known linear pyrazinone precursors which cyclize to pyrazinones, we suspect that *in vivo* conditions promote a higher level/more rapid cyclization of *N*-alanyl-aminoacetone to DPO (Figure 2E).^10^

To acquire further evidence for our suggested biosynthetic route to DPO, we examined the influence of tRNA^Ala^ on DPO production. Our rationale is that AlaRS can either use Ala-AMP to charge a tRNA^Ala^ or Ala-AMP can condense with aminoacetone and be converted to DPO. Thus, inclusion of tRNA^Ala^ in the reaction should appropriate Ala-AMP for tRNA charging, thereby decreasing its availability for DPO production. Indeed, addition of tRNA^Ala^ to the *in vitro* reaction reduced Lux bioassay activity by 60%. By contrast, addition of an aminoacylation deficient tRNA (tRNA^AlaU3:G70^)^19^ did not alter Lux bioassay activity (Figure S2A). These data suggest that tRNA^Ala^ and DPO compete for the shared intermediate, Ala-AMP. Further supporting this interpretation are data from *in vitro* reactions carried out with an AlaRS aminoacylation mutant protein (AlaRS 368N) (Figure S2B). AlaRS 368N is a truncated protein that harbors the adenylation domain but lacks the tRNA charging domain. Thus, AlaRS 368N can activate alanine with wildtype (WT) efficiency but cannot charge tRNAs.^20,21^ *In vitro* reactions with AlaRS 368N yielded the same amount of Lux bioassay activity and activated alanine with the same efficiency as the WT AlaRS enzyme (Figure 2A,B,F,G). Moreover, inclusion of either tRNA^Ala^ or tRNA^AlaU3:G70^ did not affect Lux bioassay activity in reactions catalyzed by AlaRS 368N (Figure S2B). Finally, the reactions yielded similar amounts of ^13^C_3_-*N*-alanyl-aminoacetone over time as was made with WT AlaRS and it cyclized to ^13^C_3_-DPO at a similar rate (Figure 2H-J). These results show that Ala-AMP is a DPO intermediate and that the AlaRS adenylation domain is sufficient for DPO production and the tRNA charging domain is dispensable.

Adenylation of alanine is a prerequisite for its condensation with aminoacetone to form *N*-alanyl-aminoacetone which cyclizes to DPO. One possibility is that once Ala-AMP is formed, it is released from AlaRS, enabling it to spontaneously condense with aminoacetone and then cyclize to produce DPO. Indeed, earlier work shows that *in vitro* production of linear pyrazinone precursors catalyzed by tRNA-synthetases increases over multiple hours, suggesting that the aminoacyl-adenylate reacts spontaneously with aminoacetone.^10^ If so, in our case, incubating Ala-AMP with aminoacetone should produce *N*-alanyl-aminoacetone. Unfortunately, we cannot perform this simple and obvious experiment because Ala-AMP is highly unstable. Thus, no dependable synthetic procedure exists to make it, nor can Ala-AMP be purchased. To gain initial insight into *N*-alanyl-aminoacetone condensation, we performed *in vitro* reactions with AlaRS and AlaRS 368N and measured ^13^C_3_-*N*-alanyl-aminoacetone and PP_i_ from 30 min to 4 h. ^13^C_3_-*N*-alanyl-aminoacetone and PP_i_ production by AlaRS and AlaRS 368N increased linearly over the time course and resulted in 10% and a 16% yields, respectively after 4 h (Figures 2D,I, S3A-D). Again, there was a 1:1 conversion of ^13^C_3_-Ala-AMP to ^13^C_3_-*N*-alanyl-aminoacetone. These data show that the rate of alanyl-adenylate formation sets the rate of the condensation reaction. Thus, AlaRS may not play any role beyond providing the Ala-AMP intermediate to DPO.

Ala-AMP is one of several modified forms of alanine used by bacteria. To determine if other forms of alanine can be employed as an intermediate in DPO production, we performed *in vitro* reactions substituting d-alanyl-d-ala or d-ala-phosphate for Ala-AMP. d-alanyl-d-ala is a component of peptidoglycan and d-ala-phosphate is the intermediate in d-alanyl-d-ala production. In the above *in vitro* reactions, we used l-Ala, the natural substrate for AlaRS. However, the stereochemistry of alanine is not relevant to the final stereochemistry of DPO, and alanine stereochemistry does not affect the activity of *N*-alanyl-aminoacetone.^5,11^ d-alanyl-d-ala is commercially available while d-ala-phosphate is not. To obtain d-ala-phosphate for *in vitro* reactions, we purified the d-alanine ligase B enzyme (DdlB) and incubated it with ATP, aminoacetone, ^13^C_3_-d-alanine (^13^C_3_-d-Ala), and pyrophosphatase to produce ^13^C_3_-d-ala-phosphate.^22^ Reactions with aminoacetone and synthetic d-alanyl-d-ala or ^13^C_3_-d-ala-phosphate did not result in activity in the Lux bioassay and UPLC-MS confirmed that neither ^13^C_3_-*N*-alanyl-aminoacetone nor ^13^C_3_-DPO was produced (Figure S4A–F). While it remains possible that some other activated alanine moiety or moieties may be a DPO intermediate, here, we continue to focus on Ala-AMP because it is the only verified DPO intermediate containing alanine that we know.

### Multiple Ala-AMP Producing tRNA Synthetase Enzymes Can Produce DPO If Aminoacetone Is Provided

Our above data suggest that a key step in DPO biosynthesis is activation of alanine to provide the needed Ala-AMP intermediate, which we suggest spontaneously condenses with aminoacetone to form *N*-alanyl-aminoacetone, which then cyclizes to DPO. An alternative possibility is that once formed, Ala-AMP stays bound to AlaRS with AlaRS acting as a scaffold to promote the condensation steps. In the former case, other tRNA synthetases capable of producing and releasing Ala-AMP should be able to substitute for AlaRS in the *in vitro* reaction. In the latter case, DPO production should be exclusive to AlaRS. To explore these possibilities, we tested other Ala-AMP producing tRNA synthetase enzymes for the capacity to make *N*-alanyl-aminoacetone.

tRNA synthetases are grouped into structural classes and subclasses. AlaRS is a member of the class II tRNA synthetase family that harbor a unique α + β fold that is present in few other proteins.^23–25^ Conversely, the catalytic domains of class I tRNA synthetases feature a Rossman fold and the conformations their substrates adopt for binding differ from those used to bind to class II tRNA synthases.^26–28^ To determine if Ala-AMP production or the class II structural fold of the tRNA synthetase is the key for DPO production, we performed *in vitro* reactions with two purified class II tRNA synthases, prolyl- and seryl-tRNA synthases (ProRS and SerRS), and two class I synthases, valyl- and tyrosyl-tRNA synthetases (ValRS and TyrRS). We chose these four enzymes because ProRS and ValRS, although members of different structural classes, are both promiscuous and often mis-activate alanine. One pathway for ProRS editing of mis-activated alanine is via release of Ala-AMP.^29^ In the case of ValRS, the activation site can exclude amino acids with side chains larger than that of its cognate amino acid valine, but not amino acids with smaller side chains, including alanine, so such non-cognate amino acids are adenylated.^30^ Furthermore, Ala-AMP likely has a lower affinity for the ValRS active site and may dissociate faster than Val-AMP. SerRS and TyrRS, by contrast, have stringent active sites that discriminate against non-cognates including alanine.^31,32^ Thus, mis-activation of alanine by SerRS or TyrRS is unlikely.

When ^13^C_3_-l-Ala, ATP, aminoacetone, and pyrophosphatase were provided, ProRS and ValRS produced activity as judged by the Lux bioassay, and UPLC-MS confirmed that ^13^C_3_-*N*-alanyl-aminoacetone was produced (Figure 3A–C). As with AlaRS and AlaRS 368N, ValRS drove a 1:1 conversion of ^13^C_3_-Ala-AMP to ^13^C_3_-*N*-alanyl-aminoacetone. Regarding ProRS, 4-times more PP_i_ than ^13^C_3_-*N*-alanyl-aminoacetone was produced (Figure 3B,C). Unequal amounts of the two reaction products are likely due to ProRS-catalyzed editing that hydrolyzes mis-activated Ala-AMP.^33^ Thus, only a fraction (in our case, one-fourth) of the ^13^C_3_-Ala-AMP produced by ProRS survives to contribute to ^13^C_3_-*N*-alanyl-aminoacetone production. Like AlaRS and AlaRS 368N, ^13^C_3_-*N*-alanyl-aminoacetone production by ProRS and ValRS increased linearly over the time course (1.5% and 20% yields, respectively after 4 h), indicative of a spontaneous reaction mechanism in which released Ala-AMP condenses with aminoacetone (Figure 3D). Our controls show that omission of enzyme or ATP eliminated activity in the Lux bioassay (Figure S5A–D). Neither ^13^C_3_-Ala-AMP nor ^13^C_3_-*N*-alanyl-aminoacetone could be detected in reactions with SerRS and TyrRS. We verified that both enzymes are functional as they produced their respective adenylates when provided with their cognate amino acids (l-serine for SerRS and l-tyrosine for TyrRS, Figure S5E).

**Figure 3:**
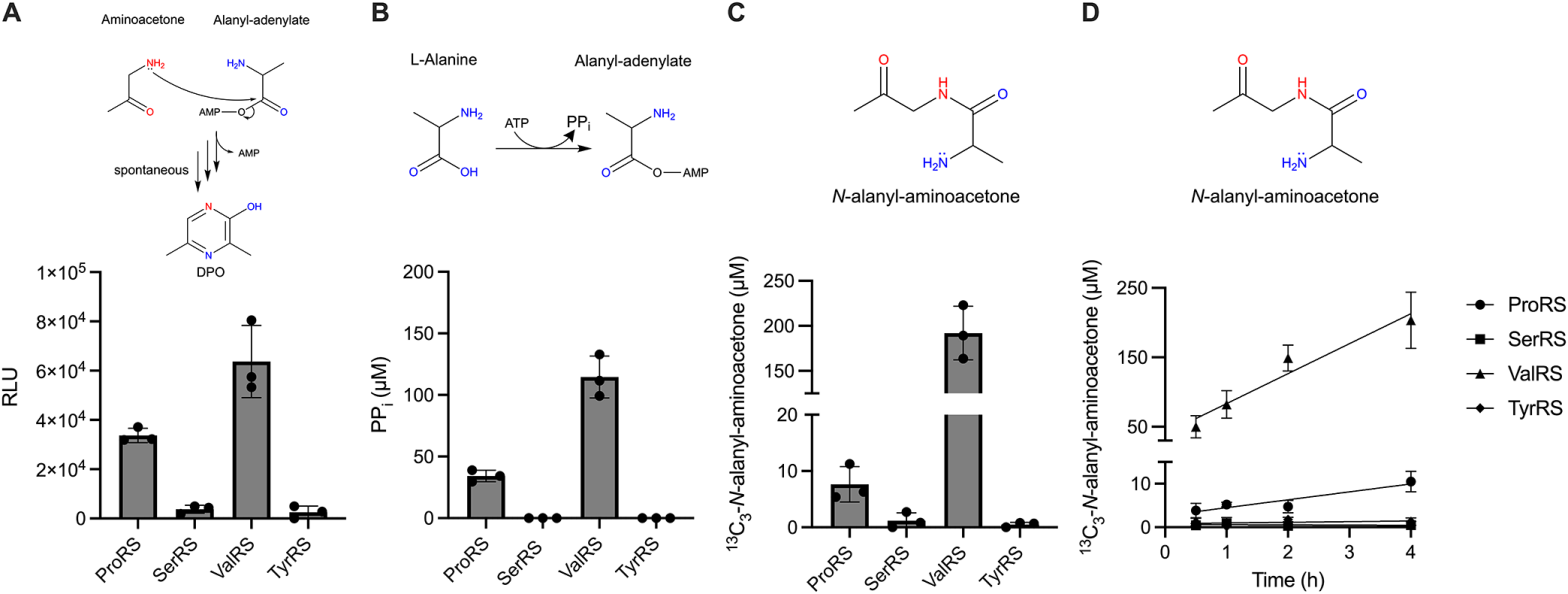
Multiple Ala-AMP producing tRNA synthetase enzymes can make *N*-alanyl-aminoacetone, and thus DPO, if aminoacetone is provided. *In vitro* reactions were carried out as in Figure 2 with the ProRS, SerRS, ValRS, or TyrRS enzymes. (A) Quantitation of activity in the Lux bioassay. RLU denotes relative light units. (B) Quantitation of PP_i_ production as assessed by the malachite green assay. (C, D) Quantitation of ^13^C_3_-*N*-alanyl-aminoacetone as assessed by UPLC-MS at 2 h (C) and over the designated time course (D). See Figures S1B,C for P_i_ and *d*_3_-*N*-alanyl-aminoacetone UPLC-MS standard curves, respectively. See Figure S5A,B for controls. See Figure S5C,D for quantitation of time course Lux bioassay activity and PP_i_ production, respectively. [PP_i_] is a proxy for [^13^C_3_-Ala-AMP] (see legend to Figure 2B). In panels A-B, reactions were normalized to samples lacking enzyme. Linear regression analysis was used to fit the data (solid lines); the R-squared values for ValRS and ProRS are 0.6940 and 0.8430, respectively (D). In all panels, error bars denote standard deviations of three biological replicates. The reaction step or product measured is shown above each panel. Red and blue heteroatoms denote, respectively, the atoms contributed by threonine and alanine to DPO.

Collectively, these data suggest that the proclivity for Ala-AMP production is the only predictor of whether a tRNA synthetase will make DPO and thus, the particular structural class and substrate binding conformations play no roles. Given the variety of classes of tRNA synthetases capable of participating in DPO production, our data suggest their only function is to supply the Ala-AMP intermediate. Thus, we conclude that AlaRS is not required to act as a scaffold to promote the later condensation and cyclization steps with aminoacetone.

### Multiple Ala-AMP Producing Enzymes Can Make DPO If Aminoacetone Is Provided

The range of tRNA synthetase enzymes capable of producing DPO show that AlaRS is not singular, so perhaps, neither are tRNA synthetases. Conceivably, any enzyme that can activate alanine to produce Ala-AMP could provide the intermediate required for DPO production if aminoacetone is available. Indeed, we hypothesize that combining Ala-AMP and aminoacetone in the absence of any enzyme would produce *N*-alanyl-aminoacetone and thus DPO. Again, Ala-AMP is unstable, not commercially available, and there is no reliable synthetic procedure. Therefore, we cannot perform this definitive experiment to test our prediction. To circumvent this issue, we tested if adenylate-forming enzymes that are not tRNA synthetases can also produce *N*-alanyl-aminoacetone and thus DPO if we provide them aminoacetone. We purified two carrier protein acyl-ligases and examined their abilities to produce DPO.

First, the adenylate-forming enzyme d-alanine-d-alanyl carrier protein ligase (DltA) from *Bacillus subtilis*: DltA produces d-Ala-AMP as an intermediate in the d-alanylation of lipoteichoic acids in Gram-positive bacteria. Following catalysis, DltA ligates d-Ala onto the carrier protein DltC with concomitant release of AMP. DltC, DltB, and DltD subsequently append d-Ala to lipoteichoic acids.^34^ Again, because the stereochemistry of alanine is not relevant to the final stereochemistry of DPO and stereochemistry does not affect the activity of *N*-alanyl-aminoacetone,^5,11^ we used the native substrate d-Ala for reactions with DltA.

Second, the adenylate-forming enzyme AlmE from *V. cholerae*: AlmE catalyzes the aminoacyl esterification of l-glycine (l-Gly) or diglycine onto lipid A, producing l-Gly-AMP as an intermediate. Following catalysis, AlmE ligates l-Gly onto the carrier protein AlmF with concomitant release of AMP. AlmG subsequently transfers the glycine from AlmF onto the lipid A acyl chain.^35^ Analogous to ProRS and ValRS, AlmE is promiscuous, and can mis-activate d-Ala. Interestingly, if AlmE makes d-Ala-AMP, it cannot be transferred to the next carrier protein, AlmF, potentially generating a pool of d-Ala-AMP.^36^

Reaction mixtures containing DltA or AlmE, ATP, aminoacetone, ^13^C_3_-d-Ala, and pyrophosphatase produced activity as judged by the Lux bioassay, and UPLC-MS confirmed production of ^13^C_3_-*N*-alanyl-aminoacetone. Like all other enzymes tested here, ^13^C_3_-*N*-alanyl-aminoacetone production increased linearly over the time course (20% and 4.5% yields, respectively) after 4 h (Figure 4A–D). Again, omission of ATP or enzyme eliminated Lux bioassay activity (Figure S6A–D). DltA- and AlmE-driven ^13^C_3_-Ala-AMP conversion to ^13^C_3_-*N*-alanyl-aminoacetone mirrored reactions with AlaRS, AlaRS 368N, and ValRS. However, we note that the P_i_ signal from reactions with AlmE were suppressed due to the necessary addition of glycerol to enzyme preparations.^37^ In contrast to the tRNA synthetases tested, addition of pyrophosphatase to reactions with DltA had only a modest effect, increasing ^13^C_3_-*N*-alanyl-aminoacetone production by ∼2-fold, and pyrophosphatase did not alter production by AlmE (Figure S6A), presumably because DltA and AlmE are better than tRNA synthetases at discriminating between ATP and PP_i_.

**Figure 4:**
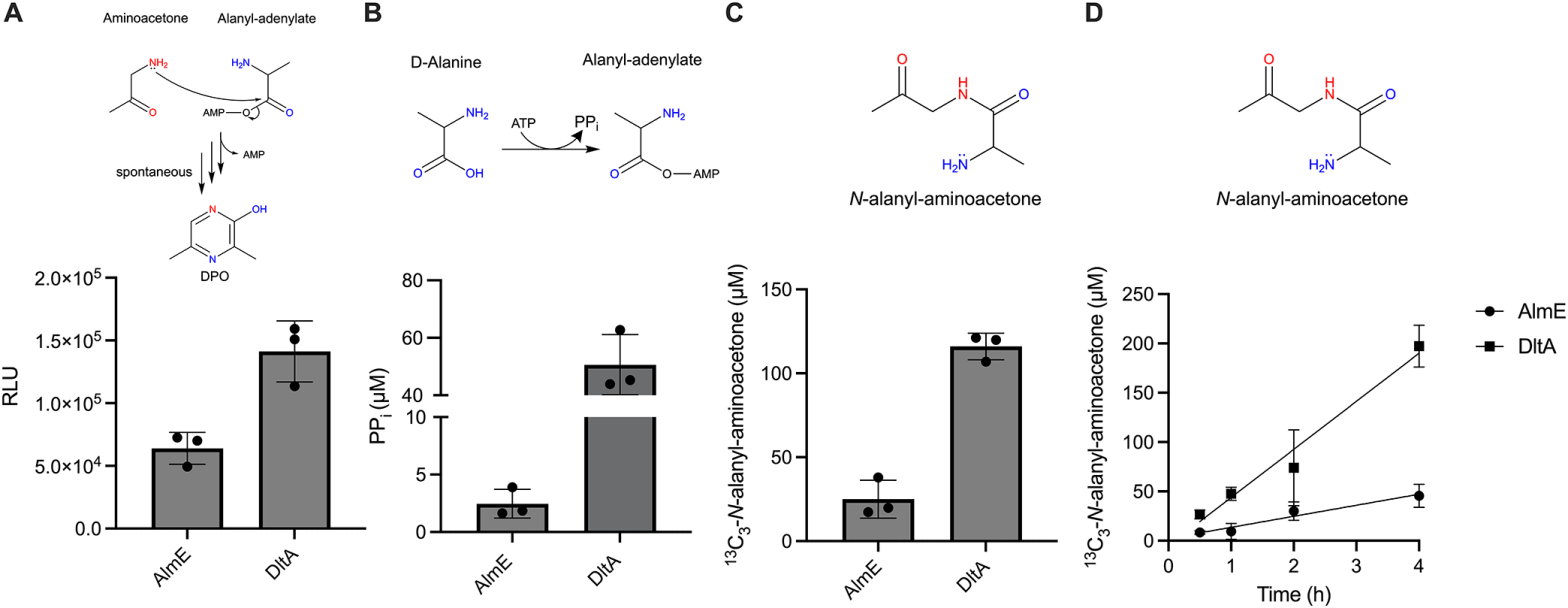
Multiple Ala-AMP producing enzymes that are not tRNA synthetases can make DPO. *In vitro* reactions were carried out as in Figure 2 with AlmE or DltA and ^3^C_3_-d-Ala. (A) Quantitation of activity in the Lux bioassay. RLU denotes relative light units. (B) Quantitation of PP_i_ production as assessed by the malachite green assay. (C, D) Quantitation of ^13^C_3_-*N*-alanyl-aminoacetone as assessed by UPLC-MS at 2 h (C) and over the designated time course (D). See Figures S1B,C for P_i_ and *d*_3_-*N*-alanyl-aminoacetone UPLC-MS standard curves, respectively. See Figures S6A,B for controls. See Figure S6C,D for quantitation of time course Lux bioassay activity and PP_i_ production, respectively. [PP_i_] is a proxy for [^13^C_3_-Ala-AMP] (see legend to Figure 2B). In panels A and B, reactions were normalized to samples lacking enzyme. Linear regression analysis was used to fit the data (solid lines); the R-squared values for DltA and AlmE are 0.9048 and 0.7867, respectively (D). In all panels, error bars denote the standard deviations of three biological replicates. The reaction step or product measured is shown above each panel. Red and blue heteroatoms denote, respectively, the atoms contributed by threonine and alanine to DPO.

Our data demonstrate that five different adenylate forming enzymes, with distinct biological functions, structures, species of origin, and amino acid affinities yield *N*-alanyl-aminoacetone, the linear precursor to the DPO autoinducer. What unifies these enzymes is that they all make Ala-AMP. We conclude that, unlike all other known QS autoinducers, perhaps there is no dedicated DPO synthetase, rather, many enzymes contribute to its production *in vivo*.

### *In vivo* DPO Production Increases When Exogenous Threonine Is Supplied

DPO biosynthesis requires two amino acids (threonine and alanine), two enzyme-catalyzed reactions (Tdh-driven and adenylate-forming enzyme-driven), one spontaneous condensation reaction (aminoacetone with Ala-AMP), and a cyclization step (*N*-alanyl-aminoacetone to DPO) that is likely promoted by *in vivo* conditions. Presumably, formation of aminoacetone and Ala-AMP are rate limiting as they depend on the availability of the amino acid substrates and the catalytic activities of the two enzymes. We have previously shown that externally supplied threonine, but not alanine, increases DPO production.^5^ To explore whether threonine is rate limiting for DPO production, we grew WT *V. cholerae* or *E. coli* in minimal medium supplemented with different concentrations of l-threonine (l-Thr), collected cell-free culture fluids, and measured activity using our Lux bioassay.

Lux bioassay activity made by both *V. cholerae* and *E. coli* increased in dose-dependent manners with increasing exogenous l-Thr (Figure 5A). Thus, fluctuations in external threonine may regulate DPO production by modulating the available pool of cytoplasmic aminoacetone, the threonine-derived DPO precursor. Indeed, DPO production by *V. cholerae* and *E. coli* cultures at high-cell density occurs as soon as 15 min after addition of l-Thr (Figure 5B), suggesting that cells can rapidly alter DPO production, and thus QS output, in response to changes in environmental threonine.

**Figure 5:**
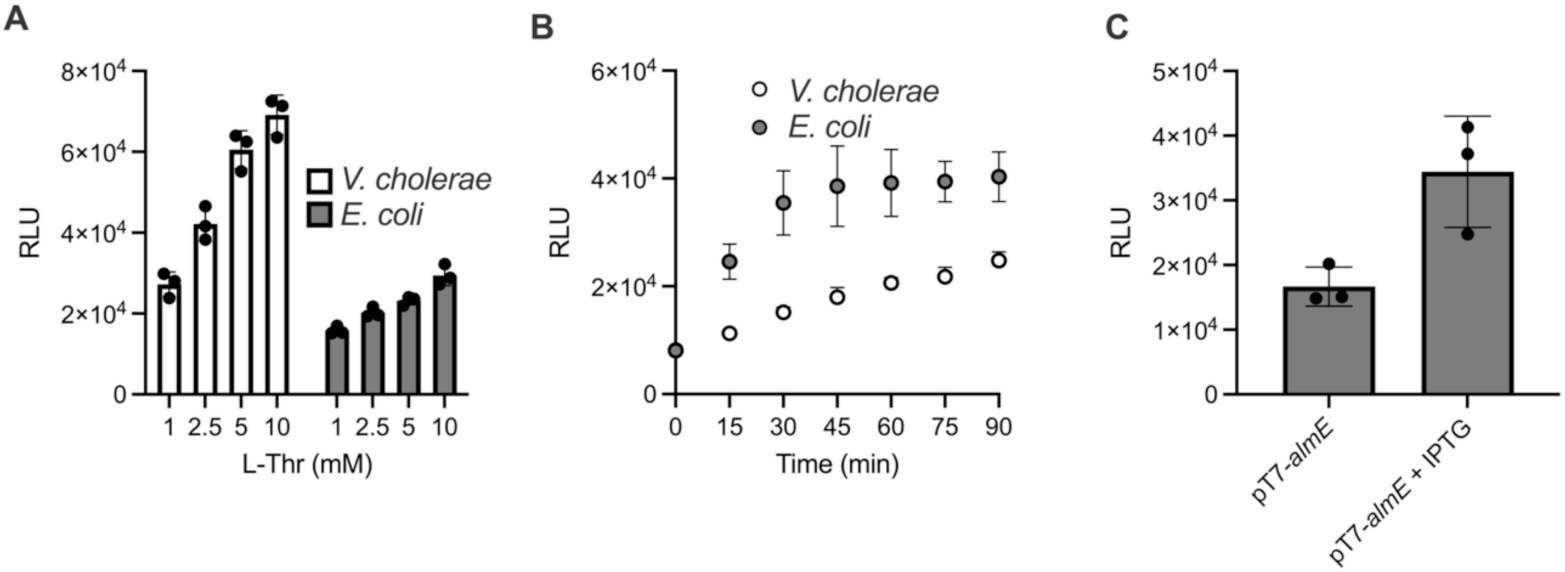
*In vivo* DPO activity increases in response to administration of external threonine. (A) Lux bioassay quantitation of activity in cell-free culture fluids of WT *V. cholerae* and *E. coli* strains supplemented with the designated concentrations of l-Thr. (B) Time course of Lux bioassay activity made by WT *V. cholerae* and *E. coli* following addition of 10 mM l-Thr. (C) *E. coli* harboring a vector carrying IPTG-inducible *almE* supplemented with 2.5 mM l-Thr with or without 1 mM ITPG. RLU denotes relative light units. In all panels, error bars denote standard deviations of three biological replicates.

In addition to availability of environmental threonine, the particular levels of adenylate-forming enzymes present in cells could affect DPO production. To test this possibility, we overexpressed *V. cholerae almE* in *E. coli* grown in minimal medium supplemented with l-Thr. Importantly, *E. coli* does not encode an AlmE enzyme. Overexpression of *almE* doubled the activity produced (Figure 5C). By contrast, the activity produced in the Lux bioassay in response to cell-free culture fluids from the Δ*almE V. cholerae* strain was not different from that made by WT *V. cholerae* (Figure S7A). Together, these data suggest that DPO production is sensitive to changes in external threonine abundance and that increasing the pool of enzymes capable of making DPO can increase production if sufficient threonine is present, whereas removing a single adenylate-forming enzyme, at least in the case of AlmE and *V. cholerae*, does not have an effect, presumably due to redundancy in the set of adenylate-forming enzymes participating in DPO production.

## Discussion

The QS autoinducer DPO is produced from aminoacetone via the action of the conserved enzyme threonine dehydrogenase (Tdh). Specifically, Tdh converts l-threonine to 2-amino-3- ketobutyric acid which, following spontaneous decarboxylation, yields aminoacetone. Aminoacetone spontaneously condenses with alanyl-adenylate (Ala-AMP) to yield *N*-alanyl-aminoacetone, which cyclizes to form DPO. Crucially, we show here that the needed Ala-AMP is derived from multiple adenylate-forming enzymes. Indeed, at least five adenylate-forming enzymes can make, release, and supply the Ala-AMP intermediate required for *N*-alanyl-aminoacetone biosynthesis *in vitro*. It is possible that other amino acid derived linear precursors to pyrazinones also use adenylates as intermediates.^10^ We find that *N*-alanyl-aminoacetone spontaneously cyclizes to form DPO *in vitro*, yet we and others have not detected *N*-alanyl-aminoacetone *in vivo*.^10^ One possibility is that *in vivo* conditions promote a higher level or more rapid cyclization of *N*-alanyl-aminoacetone to DPO than *in vitro* settings. Indeed, the addition of sucrose, a crowding agent, accelerated *N*-alanyl-aminoacetone/DPO production in a dose-dependent manner as assessed by activity in the Lux bioassay (Figure S8A). Addition of sucrose also increased Ala-AMP formation by the five adenylate-forming enzymes tested here (Figure S8B). Crowding is known to improve the catalytic efficiencies of some enzymes, including tRNA synthetases.^38^ Thus, the crowded cellular environment likely fosters both the catalytic and the spontaneous steps in DPO production. Nonetheless, given that *N*-alanyl-aminoacetone activates the VqmA receptor with similar potency as DPO, there could be particular cellular conditions where *N*-alanyl-aminoacetone persists and functions as an autoinducer.

Consistent with our findings, introduction of the AlmE adenylate-forming enzyme into *E. coli*, which does not naturally possess AlmE, increased DPO activity *in vivo* whereas deletion of *almE* from *V. cholerae*, which possesses multiple other enzymes that can compensate for its loss, did not affect DPO activity (Figures 5C and S7A). We propose that, unlike all other known QS autoinducers, perhaps there is no dedicated DPO synthase, but rather, a collection of enzymes contribute to DPO production *in vivo*. Such a mechanism implies redundancy of function and would explain the failure of traditional mutagenesis approaches to reveal genes other than *tdh* to be involved in DPO production.^5,10^

Other important signaling molecules are also produced by the collective contributions of multiple enzymes. For example, the putative alarmone and second messenger molecule adenosine tetraphosphate (Ap4a), results from the condensation of any one of a suite of adenylate molecules and ATP. Ap4a can be produced by tRNA synthetases, DNA and RNA ligases, acyl-coenzyme A synthetases, and firefly luciferase.^39–42^ *E. coli* undergoing exponential growth has been reported to produce 0.2–1 μM Ap4A,^43,44^ a concentration in line with what we measure for DPO at stationary phase in *V. cholerae* and in *E. coli* (Figure 5A). Analogous to DPO, adenylate-forming enzymes produce Ap4A with different efficiencies.^45^

Given that Ap4A and DPO are synthesized by similar mechanisms and overlapping sets of enzymes, regulators of Ap4A production could also affect DPO production. Exposing *E. coli* to a sublethal concentration of kanamycin, an inhibitor of ribosome function, increases Ap4A levels 20-fold.^46^ Ribosome inhibition coincides with the production of hydroxyl radicals.^47^ When challenged with hydrogen peroxide, which drives hydroxyl radical formation, Ap4A production increases 40-fold in *E. coli*.^46^ Administration of sublethal concentrations of erythromycin, another ribosome inhibitor, modestly increased tRNA synthetase-dependent pyrazinone production in *E. coli*,^10^ and as described, DPO is a pyrazinone. Indeed, treatment with erythromycin caused a similar minor increase in DPO activity ∼1.5-fold and ∼2-fold in *V. cholerae* and in *E. coli*, respectively (Figure S7B). Thus, preliminarily, slowed translation elongation, as a consequence of encountering erythromycin, may increase the pool of available Ala-AMP for DPO production.

The ability of the AlmE enzyme to contribute to DPO production is especially intriguing. AlmE has been hypothesized to participate in the biosynthesis of some unidentified compound that affects *V. cholerae* biofilm formation.^48^ We propose that this putative compound is DPO because, in *V. cholerae*, DPO bound to its partner VqmA receptor modulates biofilm development. Specifically, the DPO-VqmA complex activates expression of *vqmR*, encoding the VqmR regulatory small RNA. VqmR represses translation of *vpsT.* VpsT activates expression of multiple genes required for biofilm formation including *vpsL*, the first gene in the *vpsII*-operon responsible for synthesis of vibrio polysaccharide (VPS). VPS is the major polysaccharide component of the *V. cholerae* biofilm matrix. Thus, DPO production represses *vpsL* expression which, in turn, suppresses biofilm formation. Consistent with this mechanism, Δ*almE V. cholerae* strains display enhanced biofilm formation and increased *vpsL* expression. Furthermore, deletion of *almF* or *almG* does not affect biofilm formation nor *vpsL* expression while the triple Δ*almEFG* mutant phenotype is identical to that of the Δ*almE* mutant.^48^

In *V. cholerae, almE* expression is activated by the two-component CarRS system, which responds to changes in external Ca^2+^ concentrations. *carRS* transcription is repressed in the presence of high Ca^2+^.^48,49^ Concentrations of Ca^2+^ are high in the marine environment compared to that in the human gut.^50,51^ Thus, our expectation is that *almE* will be more highly expressed when *V. cholerae* is in the human host compared to when it is in the ocean. Based on our finding that AlmE contributes to DPO production, and DPO inhibits *V. cholerae* biofilm formation, we suggest that AlmE could promote *V. cholerae* dissemination from the host back to the marine niche.

The involvement of multiple adenylate-forming enzymes in DPO biosynthesis presents new possibilities for the regulation of information flow through a QS pathway. Take for example, tRNA synthetases: they are universal, highly regulated, and play essential roles in protein translation. Their activities can be modulated through amino acid starvation, tRNA aminoacylation levels, ribosome activity, and cellular growth rates.^12^ Likewise, the additional participating adenylate-forming enzymes are subject to regulation by other inputs (i.e., AlmE and Ca^2+^). Going forward, it will be fascinating to explore how perturbations in protein synthesis or other relevant stimuli affect DPO production.

The release of Ala-AMP by adenylate-forming enzymes appears to be critical for DPO production. Presumably, adenylate-forming enzymes for which alanine is a non-cognate substrate will have a lower affinity for the Ala-AMP product than enzymes that have evolved to use alanine to form an adenylate. Thus, the former could be more prone to Ala-AMP release. Indeed, ValRS prefers valine over alanine, and yet was the most avid DPO producer among the set of enzymes tested here (Figure 3). Editing activities of tRNA synthetases, like that of ProRS, which eject mis-activated Ala-AMP in order to accommodate the correct amino acid for tRNA charging, may also promote DPO production.^29^

Beyond regulation of DPO production through control of adenylation activity, the requirement for threonine could also connect particular environmental conditions to QS output. Indeed, DPO activity increases with increasing exogenous l-threonine in both *V. cholerae* and *E. coli* (Figure 5A). By contrast, addition of alanine does not influence DPO activity.^5^ These findings are consistent with measurements showing that in glucose-fed exponentially growing *E. coli*, alanine is >10-fold more abundant than threonine (2.6 × 10^–3^ and 1.8 × 10^–4^ mol L^–1^, respectively).^52^ Together with our finding that Ala-AMP is supplied by adenylate-forming enzymes, basal levels of unbound Ala-AMP must be sufficient to support DPO production, while endogenous levels of threonine are not. Apparently, endogenously synthesized threonine is incapable of supporting both protein synthesis and DPO production, so protein production, a process essential for survival, is prioritized over orchestrating collective behaviors, which could be a luxury under starvation conditions.

The intestinal tract, the location of *V. cholerae* infection, is a high threonine environment due to high levels of the protein mucin, which can be >30% threonine.^53,54^ *V. cholerae* and other commensal gut bacteria encode proteases and mucinases that degrade mucin.^55,56^ Likely, access to mucin-derived threonine allows *V. cholerae* to increase DPO production in the gut, consequently, repressing biofilm formation and disseminating from the host. Thus, the absence or presence of external threonine could be the cue that alerts *V. cholerae* to undertake one of two different lifestyles – remain in the human host (threonine absence) or return to the marine niche (threonine presence). This situation parallels that concerning Ca^2+^ levels. In the gut, Ca^2+^ concentration is low, which upregulates *almE* expression,^48^ and likely DPO production. Therefore, the intestinal track, more than the ocean, may promote DPO production by *V. cholerae* due to the comparatively high concentration of threonine and low concentration of Ca^2+^.

Employing multiple synthases in the second step of DPO biosynthesis may make controlling its regulation and coordinating its accumulation with increasing cell density particularly difficult. Thus, external threonine, which directly controls the amount of DPO that will be made by controlling Tdh-directed aminoacetone production, may be the primary regulator of DPO biosynthesis. By contrast, production of Ala-AMP may tune DPO production to a few key cues such as ribosome stress. Such cues may be highly important for regulating DPO production in species other than *V. cholerae* that do not experience frequent changes in external threonine. Also, *V. cholerae* uses two other QS autoinducers, CAI-1 and AI-2, both derived from *S*-adenosyl methionine (SAM).^3,4^ The concentration of SAM is tightly regulated.^57^ Thus, cell-density dependent accumulation of CAI-1 and AI-2 may buffer QS activity from non-cell density-dependent fluctuations in DPO production.

In conclusion, biosynthesis of DPO is distinct from that of other QS autoinducers in that there is apparently no dedicated synthase. Rather, a suite of enzymes must divert resources from their established functions to contribute to the production of this QS autoinducer. The involvement of multiple adenylate-forming enzymes in DPO biosynthesis presents both new components and, importantly, new possibilities for the regulation of information flow through a QS pathway. Given that the adenylate-forming enzymes examined here are not homologous to one another, enzymes not yet identified as adenylate-forming and/or adenylate-forming enzymes that are not specific for alanine could also contribute to DPO production.

## Methods

### Bacterial Plasmids, Strains, Primers, and Reagents

Plasmids, strains, and primers/dsDNA (Ultamer duplex primer) used in this study are listed in Tables S1, S2, and S3, respectively. Unless otherwise indicated, *V. cholerae* and *E. coli* were grown aerobically in lysogeny broth (LB) at 37 °C. M9 medium was supplemented with 0.5% glucose and 0.4% casamino acids unless otherwise noted. Antibiotics and inducers were used at the following concentrations: 100 μg mL^–1^ ampicillin, 50 μg mL^–1^ kanamycin, 8 μg mL^–1^ or 2 μg mL^–1^ erythromycin for *E. coli* and *V. cholerae* respectively, 0.02% arabinose, and 1 mM Isopropyl β-D-1-thiogalactopyranoside (IPTG). Primers were obtained from Integrated DNA Technologies.

### Molecule Syntheses

^13^C-DPO and *d*_3_-*N*-alanyl-aminoacetone were synthesized by WuXi Apptec. The manufacturer estimates 100% and >95% purity, respectively, which were verified by in-house NMR analyses.

### Protein Purification

Plasmids used for production of *E. coli* AlaRS, ProRS, SerRS, ValRS, TyrRS (gifts from Cheemeng Tan, (Addgene plasmids #111457, #111486, #111487, #111463, and #111491))^58^, AlaRS 368N, and DdlB (Genescript) were transformed into *E. coli* BL21 (DE3) (NEB) grown in LB medium with appropriate antibiotics and induced with IPTG at OD_600_ 0.5-0.7. After 4 h of growth, cells were pelleted at 4,000 rpm for 10 min and resuspended in 25 mL of lysis buffer (50 mM Tris-HCl pH 8.0, 300 mM NaCl, 10 mM imidazole) with EDTA-free cOmplete UltRA Mini Protease Inhibitor Cocktail (Sigma), 1 μL of DNase 1 (NEB), and 250 μL lysozyme from egg extract (Sigma). The cells were lysed by sonication, clarified by centrifugation at 10,000 rpm for 45 min at 4 °C, and protein was purified using Ni-NTA Superflow (QIAGEN) resin. Proteins were eluted using increasing concentrations of imidazole. The fractions containing the proteins were concentrated and dialyzed into a final buffer containing 50 mM Tris-HCl pH 7.5 and 300 mM NaCl.

*B. subtilis* DltA protein (Genescript, codon optimized for *E. coli*) was produced and purified as described above with the following modifications: cells were resuspended in Tris-HCl pH 8.0, 500 mM NaCl, 10 mM imidazole and dialyzed into buffer containing 50 mM HEPES pH 7.8 and 100 mM NaCl. *V. cholerae* AlmE protein (Genescript, codon optimized for *E. coli*) was produced and purified as described above with the following modifications: induced cells were grown for 18 h at 20 °C, lysis buffer contained 20 mM HEPES pH 7.5, 500 mM NaCl, 10 mM imidazole, and 10% (v/v) glycerol, and dialysis buffer contained 150 mM NaCl, 10 mM HEPES pH 7.5, and 10% (v/v) glycerol.

### *In vitro* Reactions with Adenylate-forming Enzymes

*In vitro* reactions were carried out in aminoacylation buffer (50 mM HEPES pH 7.2, 100 mM KCl, 10 mM MgCl_2_) with 2 mM ATP (Sigma), 1 mM ^13^C_3_-l- or ^13^C_3_-d-alanine (Cambridge Isotope Laboratories), 1 mM aminoacetone (Santa Cruz Biotechnology), 0.5 U/mL yeast inorganic pyrophosphatase (Sigma), and 1 μM purified adenylate-forming enzyme. For crowding experiments, reactions were carried out in the presence of 0-5% (*w/v*) sucrose. Reactions were incubated at 37 °C for 30 min to 4 h, as indicated. Reactions were terminated by heating to 95 °C for 5 min and denatured protein was removed by centrifugation. Reactions were filtered, snap frozen with liquid nitrogen, and held at -80 °C until analysis.

### *In vitro* Reactions to Measure Conversion of *N*-alanyl-aminoacetone to DPO

*In vitro* reactions were carried out as described above with all reaction components or the reactions lacked aminoacetone. Reactions were terminated after 8 h. After termination, 200 μM, 400 μM, or 600 μM of synthetic *d*_3_-*N*-alanyl-aminoacetone was added to the reactions that did not contain aminoacetone. Reactions were subsequently incubated for 2 to 24 h at 37 °C, and the concentrations of *d*_3_-*N*-alanyl-aminoacetone and ^13^C_3_-DPO were quantified with UPLC-MS (see below).

### *In vitro* Reactions with tRNA

*E. coli* tRNA^Ala^ and tRNA^AlaU3:G70^ were synthesized according to the manufacturer’s protocol for small RNAs (HiScribe T7 High Yield RNA synthesis Kit #E2040S) using synthetic tRNA^Ala^ and tRNA^AlaU3:G70^ dsDNA oligonucleotides with a T7-promoter sequence as template (Table S3). tRNAs were purified according to the manufacturer’s protocol for small RNAs (Monarch RNA Cleanup Kit (500 μg) T2050S) and eluted in water. tRNAs were refolded by heating to 95 °C for 2 min, cooling to 20 °C for 3 min, followed by heating to 37 °C for 5 min. Samples were divided into aliquots to avoid multiple freeze-thaw cycles and held at -80 °C. tRNA^All^ was obtained from the PURExpress (Δ (aa, tRNA) Kit #E6840S, NEB). Reactions were carried out as above without pyrophosphatase and with 1 μM of tRNA^All^, tRNA^Ala^, or tRNA^AlaU3:G70^.

### Lux Bioassays to Measure DPO and *N*-alanyl-aminoacetone Activity

To measure DPO and *N*-alanyl-aminoacetone production via activation of the *V. cholerae* *vqmR-lux* reporter, overnight cultures were back-diluted 1:1000 into M9 medium with 0.02% arabinose and dispensed (200 μL) into 96-well plates (Corning Costar 3904). The plates were shaken at 37 °C and a Biotek Synergy Neo2 Multi-Mode reader was used to measure OD_600_ and bioluminescence. Relative light units (RLU) were calculated by dividing the bioluminescence values by the OD_600_ at 8 h. For *in vitro* reactions, samples were normalized to reactions lacking enzymes. Standard curves were generated using known amounts synthetic ^13^C-DPO and *d*_3_-*N*-alanyl-aminoacetone in aminoacylation buffer. To calculate expected Lux bioassay activities from ^13^C_3_-DPO and ^13^C_3_-*N*-alanyl-aminoacetone produced by *in vitro* reactions with adenylate-forming enzymes as assessed by UPLC-MS, molecule concentrations were divided by the Lux bioassay dilution factor and standard curves for each molecule were used to calculate expected RLU.

### Malachite Green Assays to Measure PP_i_, a Proxy for Aminoacyl-AMP Production

To measure PP_i_ production by adenylate-forming enzymes, *in vitro* reactions were diluted in aminoacylation buffer and dispensed (50 μL) into 96-well plates (Corning Costar 3903) containing 20 μL of malachite green reagent (Sigma) and 30 μL of water. For reactions that did not contain pyrophosphatase during incubation, 0.5 U/mL pyrophosphatase was added to terminated reactions prior to malachite green development. Plates were developed at room temperature for 30 min and OD_600_ was measured using a Biotek Synergy Neo2 Multi-Mode. Standard curves were generated with known concentrations of P_i_ or PP_i_ (Sigma) incubated with 0.5 U/mL pyrophosphatase in aminoacylation buffer for 30 min. PP_i_ is used as a proxy for aminoacyl-AMP formation, where detected [Pi]/2 equals [aminoacyl-AMP] generated. Reactions were normalized to samples lacking enzyme to account for background ATP hydrolysis.

### UPLC-MS Analysis to Measure DPO and *N*-alanyl-aminoacetone Production

All samples were analyzed by ultra-performance liquid chromatography mass spectrometry (UPLC-MS) using an Agilent 1290 Infinity II liquid chromatography system coupled to an Agilent IQ single-quadrupole mass spectrometer. Separation of molecules was achieved using a Phenomenex Synergi Polar RP column (2.5 μm, 100 Å, 3 × 100 mm), held at 30 °C. Solvent A was water with 0.1% formic acid and solvent B was methanol with 0.1% formic acid. In all cases, 5 μL of sample or standard was injected. Solvent flow rate was held at 0.3 mL min^–1^, with the column equilibrated in 25% solvent B. The solvent mixture was adjusted using a linear gradient from 25% to 64% solvent B between 1 and 6 min. For mass spectrometry, data were collected in positive mode with the capillary voltage set to 3500 V and the fragmentor set to 110 V. Nitrogen gas flow was set at 12 L min^–1^ at 325 °C with a nebulizer pressure of 50 psi. Analytes were detected at m/z values of 126.1 (^13^C_1_C_5_H_8_N_2_O, ^13^C-DPO standard), 128.1 (^13^C_3_C_3_H_8_N_2_O, ^13^C_3_-DPO product), and 148.1 (C_6_H_9_d_3_N_2_O_2_/^13^C_3_C_3_H_12_N_2_O_2_, *d*_3_-*N*-alanyl-aminoacetone standard/^13^C_3_-*N*-alanyl-aminoacetone product). Quantitation was performed using MassHunter Quantitative Analysis 11 (Agilent) to identify and calculate integrated peak areas. Manual curation confirmed peak identification and integration parameters. The standard curves for ^13^C-DPO and *d*_3_-*N*-alanyl-aminoacetone were generated by linear regression analysis and the resulting slopes used to calculate concentrations of all analytes.

### Threonine Supplementation and Overexpression of Adenylate-forming Enzymes

Cell-free culture fluids were prepared as previously described.^5^ Lux bioassay activity was determined as described above for the following growth conditions. In Figures 5A and S7A, overnight cultures of WT *V. cholerae*, Δ*almE V. cholerae*, or *E. coli* cells were back diluted 1:1000 into M9 medium supplemented with 1, 2.5, 5, or 10 mM l-Thr and grown for 12 h. In Figure 5B, following back dilution, the strains were grown to OD_600_ ∼ 1.0, at which point, 10 mM l-Thr was added. Aliquots were taken at 15 min intervals for 90 min. In Figure 5C, following back dilution of *E. coli* harboring a plasmid carrying *V. cholerae almE* under the T7 promoter, the cells were grown to OD_600_ ∼ 0.6, at which point 2.5 mM of l-Thr was added along with 1 mM IPTG or water. The cells were grown for another 7 h prior to collection of cell-free culture fluids for assessment in the Lux bioassay. In Figure S7B, following back dilution into M9 medium supplemented with 2.5 l-Thr and erythromycin (8 μg mL^–1^ or 2 μg mL^–1^ for WT *E. coli* and *V. cholerae*, respectively) or water, cells were grown for 12 h.

### Statistical Methods

All statistical analyses were performed using GraphPad Prism software. Error bars correspond to standard deviations of the means of three biological or three technical replicates as noted.

## Supplementary Material



## Notes

Table listing plasmids used; table listing strains used; table listing primers used; Lux bioassay standard curves generated with known concentrations of ^13^C-DPO and *d*_3_-*N*-alanyl-aminoacetone; malachite green assay standard curves generated with known concentrations of P_i_ or PP_i_ + pyrophosphatase; UPLC-MS standard curves generated with known concentrations of ^13^C-DPO or *d*_3_-*N*-alanyl-aminoacetone; expected Lux bioassay activity from UPLC-MS quantitation compared to actual Lux bioassay activity from *in vitro* reactions with the AlaRS and AlaRS 368N enzymes; Lux bioassay activity from *in vitro* reactions with tRNA and AlaRS or AlaRS 368N; conversion of different initial concentrations of synthetic *d*_3_-*N*-alanyl-aminoacetone to *d*_3_-DPO as assessed by UPLC-MS; time course of Lux bioassay activity and PP_i_ production by AlaRS and AlaRS 368N; *in vitro* reactions with aminoacetone and the DdlB enzyme or with d-alanyl-d-ala; controls for *in vitro* reactions with ProRS, SerRS, ValRS, TyrRS, DltA, and AlmE; Lux bioassay quantitation of activity in cell-free culture fluids of WT *V. cholerae* and Δ*almE V. cholerae* supplemented with l-Thr and WT *V. cholerae* and *E. coli* strains supplemented with l-Thr and erythromycin; *in vitro* reactions with AlaRS, ProRS, ValRS, DltA, and AlmE in the presence of 0-5% (*w/v*) sucrose (PDF)
