# Supplementary material for: Multiple Adenylate-Forming Enzymes Contribute to Biosynthesis of the DPO Quorum-Sensing Autoinducer
Source: ACS Chem Biol. 2026 Jan 30;21(2):380–91. doi: 10.1021/acschembio.5c00932 (PMC12930378; doi:10.1021/acschembio.5c00932)
Supplement: Supplementary file 1 [file cb5c00932_si_001.pdf]

Supporting Information

Multiple adenylate-forming enzymes contribute to biosynthesis of the DPO quorum-sensing autoinducer

Delaney M. Lacey[1], Gabriel D. D'Agostino[1], Emilee E. Shine[1], Bonnie L. Bassler[1, 2*]

[1]Department of Molecular Biology, Princeton University, Princeton, NJ 08544, USA

[2]Howard Hughes Medical Institute, Chevy Chase, MD 20815, USA

*To whom correspondence should be addressed: bbassler@princeton.edu

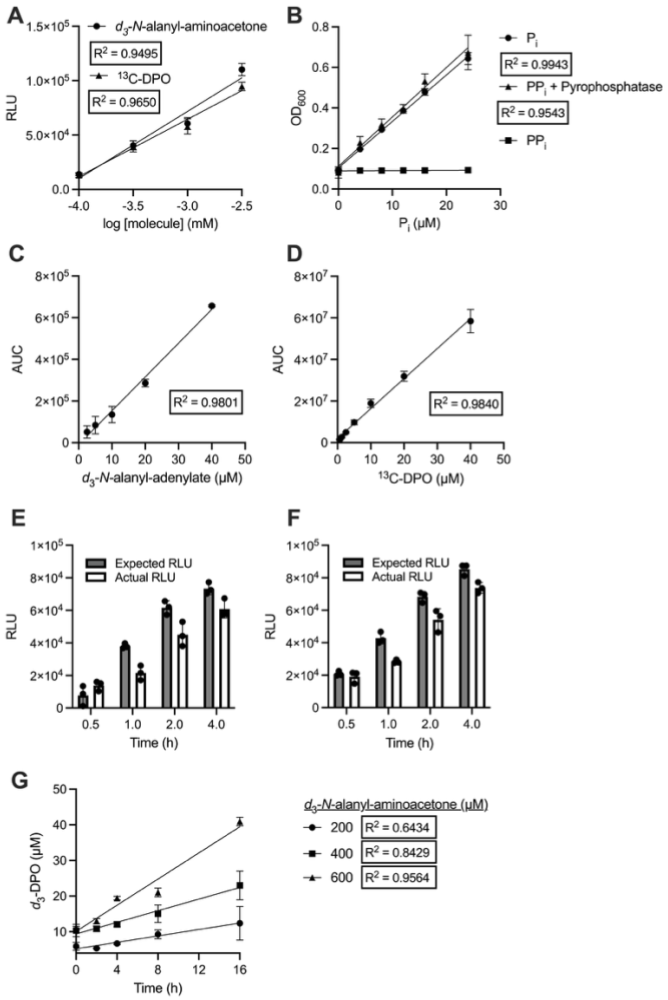

Figure S1. Standard curves for d_3-*N*-alanyl-aminoacetone, ^{13}C-DPO, and P_i. (A) Activity in the Lux bioassay for the designated concentrations of synthetic d_3-*N*-alanyl-aminoacetone and ^{13}C-DPO. RLU denotes relative light units. (B) Output from the malachite green assay showing that, under our conditions, pyrophosphatase completely hydrolyzes PP_i to P_i by the first time point (30 min) and PP_i does not produce activity. To quantify the concentrations of (C) $^{13}C_3$-*N*-alanyl-aminoacetone and (D) $^{13}C_3$-DPO in our *in vitro* reactions, UPLC-MS signals (area under the curve (AUC)) were measured for known concentrations of synthetic d_3-*N*-alanyl-aminoacetone and ^{13}C-DPO to generate the standard curves shown in these panels. Expected Lux bioassay activity from $^{13}C_3$-*N*-alanyl-aminoacetone produced in an *in vitro* reaction with the (E) AlaRS enzyme and (F) AlaRS 368N enzyme as assessed by UPLC-MS (see Figure 2D and 2I) (gray bars show expected Lux bioassay activity from UPLC-MS quantitation and white bars show actual Lux bioassay activity (see Figure S3A and S3C)). (G) Conversion of synthetic d_3-*N*-alanyl-aminoacetone to d_3-DPO from 200, 400, or 600 μM starting material, as assessed by UPLC-MS. Linear regression analyses were used to fit the data (solid lines); equations are Y = 61666*X + 256740 for d_3-*N*-alanyl-aminoacetone and Y = 52629*X + 222412 for ^{13}C-DPO(A), Y = 0.02292*X + 0.1042 for P_i and Y = 0.02438*X + 0.1118 for PP_i + Pyrophosphatase (B), Y = 16124*X – 5956 for d_3-*N*-alanyl-aminoacetone (C), and Y = 1441309*X + 1970239 for ^{13}C-DPO (D). R-squared values are indicated (A-D, G). Error bars denote standard deviations of three representative technical replicates (A-D, G) and three biological replicates (E, F).

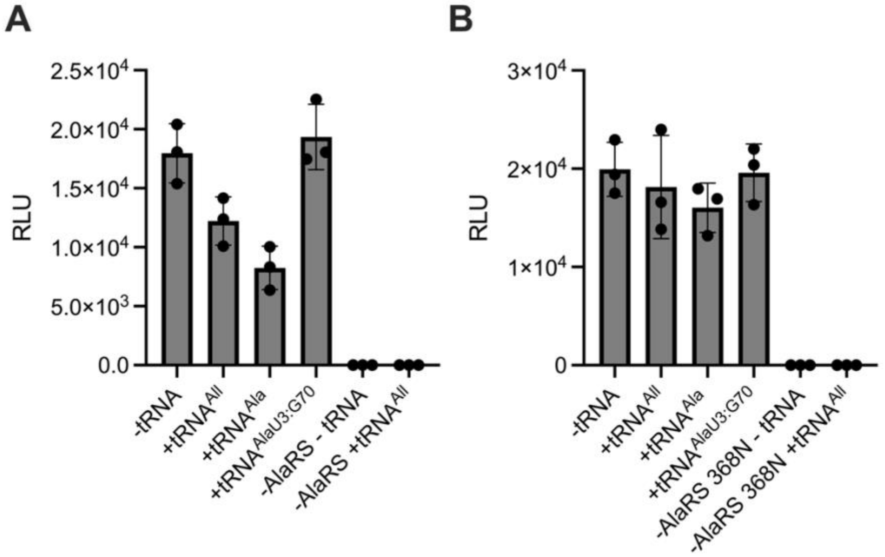

Figure S2. Inclusion of tRNA in *in vitro* reactions decreases DPO activity produced by AlaRS but does not affect DPO activity produced by AlaRS 368N. Quantitation of activity from *in vitro* reactions carried out as in Figure 2 but lacking pyrophosphatase and with (A) AlaRS or (B) AlaRS 368N enzyme and the addition of 1 μM $tRNA^{All}$ (a mixture of all 20 tRNAs), $tRNA^{Ala}$, or the aminoacylation mutant $tRNA^{AlaU3:G70}$. RLU denotes relative light units. Reactions were normalized to samples lacking enzyme. Error bars denote standard deviations of three biological replicates.

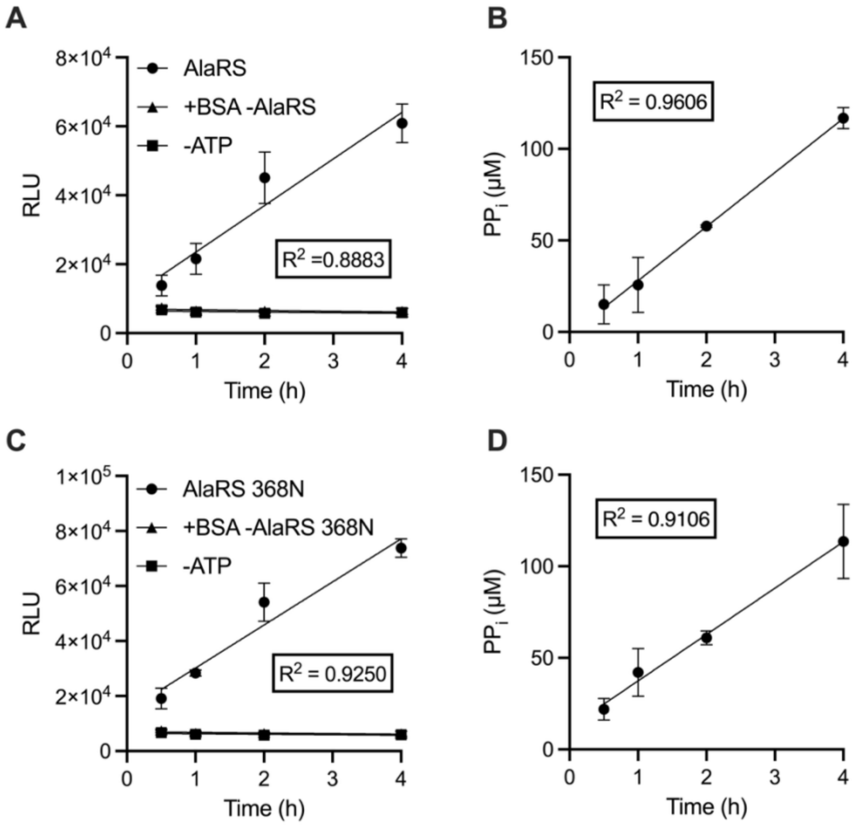

Figure S3. Lux bioassay time course and Ala-AMP production by AlaRS and AlaRS 368N. (A, C) Quantitation of activity from *in vitro* reactions carried out as in Figure 2 with the AlaRS or AlaRS 368N enzyme at the designated time points as judged by the Lux bioassay. RLU denotes relative light units. (B, D) Quantitation of PP_i production from *in vitro* reactions with the AlaRS or AlaRS 368N enzyme as assessed by the malachite green assay. In panels B and D, PP_i was determined using standard curves generated with known concentrations of P_i where $[P_i]/2$ equals $[PP_i]$ (see Figure S1B). $[PP_i]$ is a proxy for $[^{13}C_3$-Ala-AMP] (see legend to Figure 2B). All reactions were normalized to samples lacking enzyme. Linear regression analyses were used to fit the data (solid lines); the R-squared values are indicated (A-D). In all panels, error bars denote standard deviations of three biological replicates.

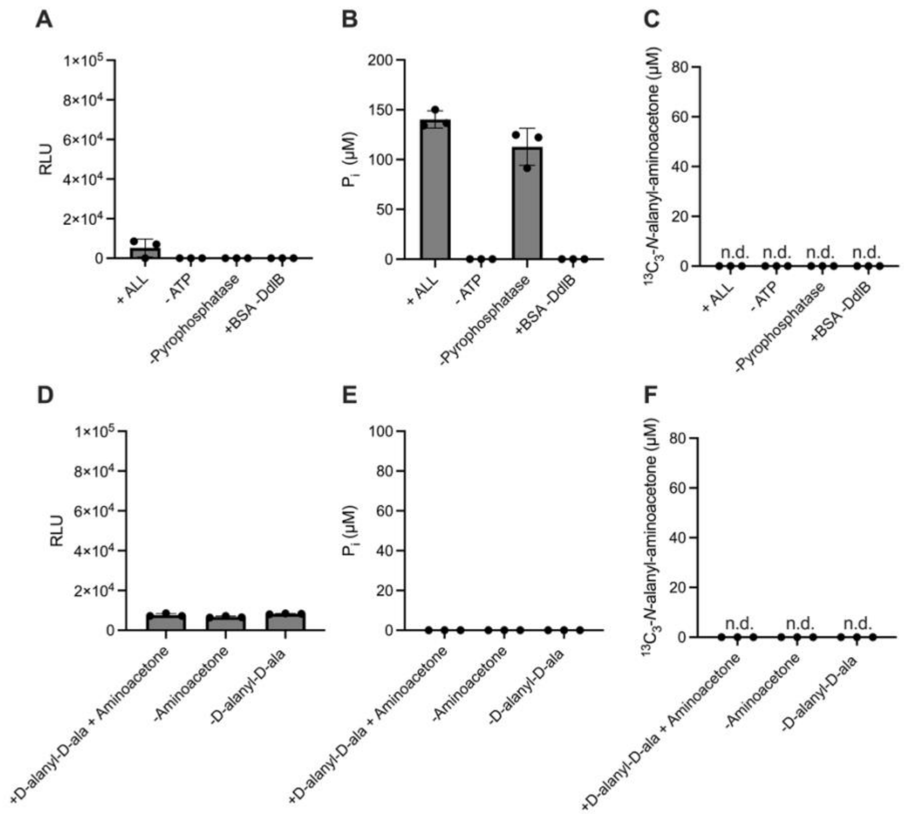

Figure S4. D-ala-phosphate and D-alanyl-D-ala are not involved in DPO production. *In vitro* reactions were carried out as in Figure 2. Reactions contained DdlB [1 µM], $^{13}C_3$-D-Ala [1 mM], aminoacetone [1mM], ATP [2 mM], and inorganic pyrophosphatase [0.5 U/mL] (A-C) or D-alanyl-D-ala [1 mM] and aminoacetone [1mM] (D-F). (A, D) Quantitation of activity in the Lux bioassay. RLU denotes relative light units. (B, E) Quantitation of P_i production from *in vitro* reactions as assessed by the malachite green assay. [P_i] is a proxy for [$^{13}C_3$-D-ala-phosphate] (1). (C, F) Quantitation of $^{13}C_3$-*N*-alanyl-aminoacetone from *in vitro* reactions assessed by UPLC-MS. In panels B and E, P_i was determined using standard curves generated with known concentrations of P_i (see Figure S1B). In panels C and F, $^{13}C_3$-*N*-alanyl-aminoacetone production was determined using standard curves generated with known concentrations of d_3-*N*-alanyl-aminoacetone (see Figure S1C). In panels A and B, reactions were normalized to samples lacking enzyme. In all panels, error bars denote standard deviations of three biological replicates.

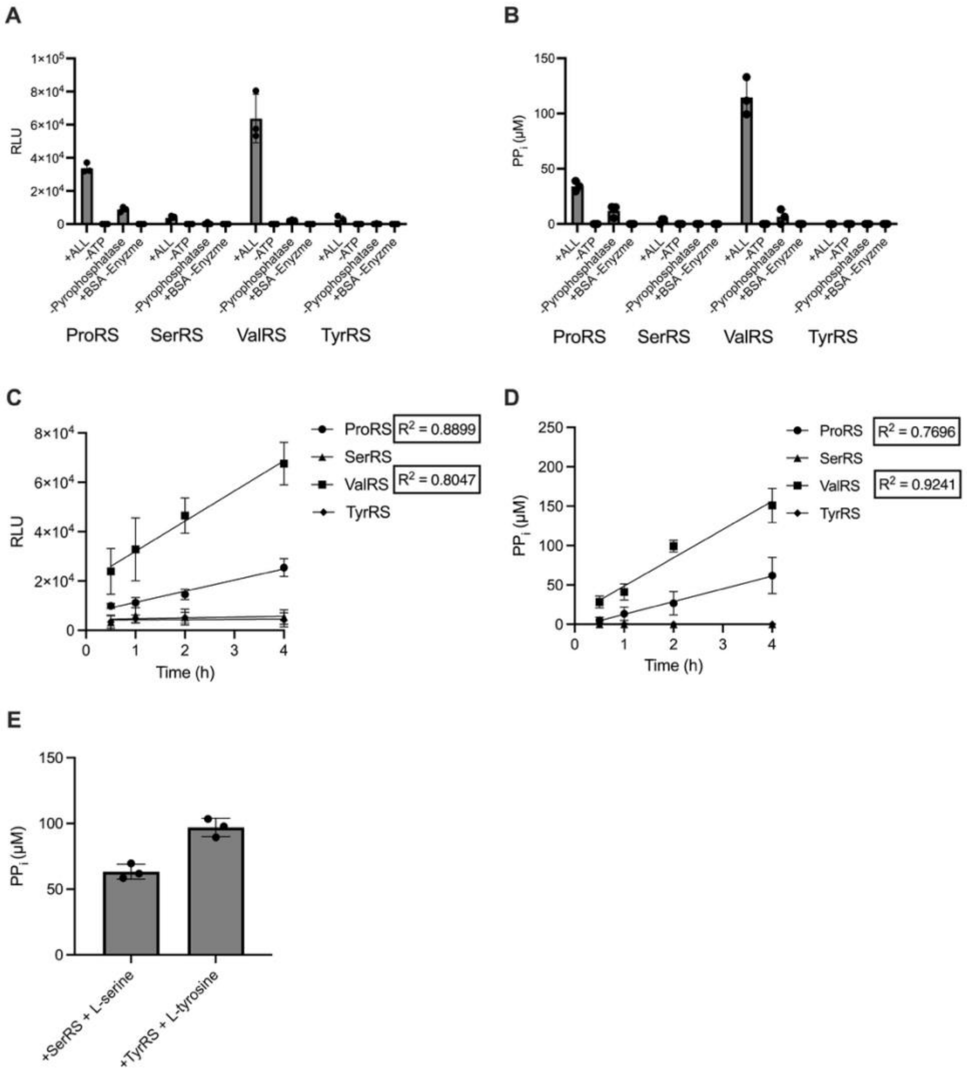

Figure S5. ATP, aminoacetone, and alanine are required for DPO production by tRNA synthetases, time courses of Lux bioassay activity, time courses of Ala-AMP production by tRNA synthetases, and SerRS and TyrRS are functional. *In vitro* reactions were carried out with as in Figure 2 with the ProRS, SerRS, ValRS, or TyrRS enzymes. (A, C) Quantitation of activity in the Lux bioassay at 2 h (A) and over the designated time course (C). RLU denotes relative light units. (B, D) Quantitation of PP_i production as assessed by the malachite green assay at 2 h (B) and over the designated time course (D). (E) Quantitation of PP_i produced by SerRS and TyrRS with their cognate amino acids, L-serine and L-tyrosine, respectively. [PP_i] is a proxy for [aminoacyl-AMP] (see legend to Figure 2B). See Figure S1B for P_i standard curves. All reactions were normalized to samples lacking enzyme. Linear regression analyses were used to fit the data (solid lines); the R-squared values are indicated (C, D). In all panels, error bars denote standard deviations of three biological replicates.

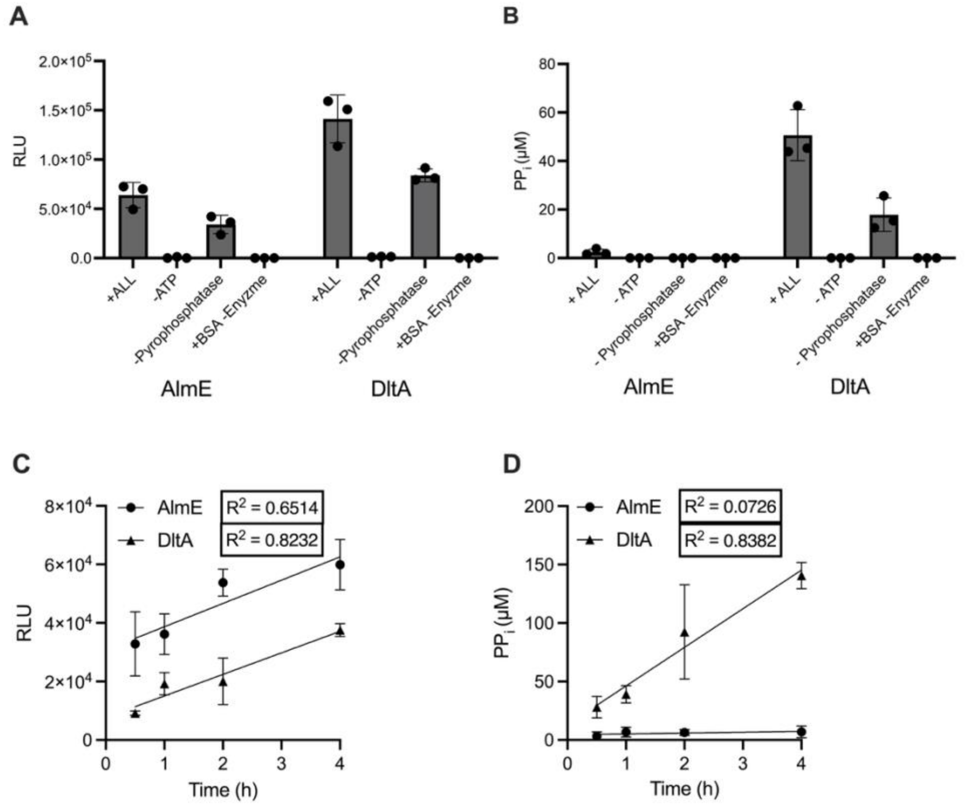

Figure S6. ATP, aminoacetone, and alanine are required for DPO production by adenylate-forming enzymes and time courses of Lux bioassay activity and Ala-AMP production by adenylate-forming enzymes. *In vitro* reactions were carried out as in Figure 2 with the AlmE and DltA enzymes. (A, C) Quantitation of activity in the Lux bioassay at 2 h (A) and over the designated time course (C). RLU denotes relative light units. (B, D) Quantitation of PP_i production as assessed by the malachite green assay at 2 h (B) and over the designated time course (D). [PP_i] is a proxy for [$^{13}C_3$-Ala-AMP] (see legend to Figure 2B). See Figure S1B for P_i standard curves. All reactions were normalized to samples lacking enzyme. Linear regression analyses were used to fit the data (solid lines); the R-squared values are indicated (C, D). In all panels, error bars denote standard deviations of three biological replicates.

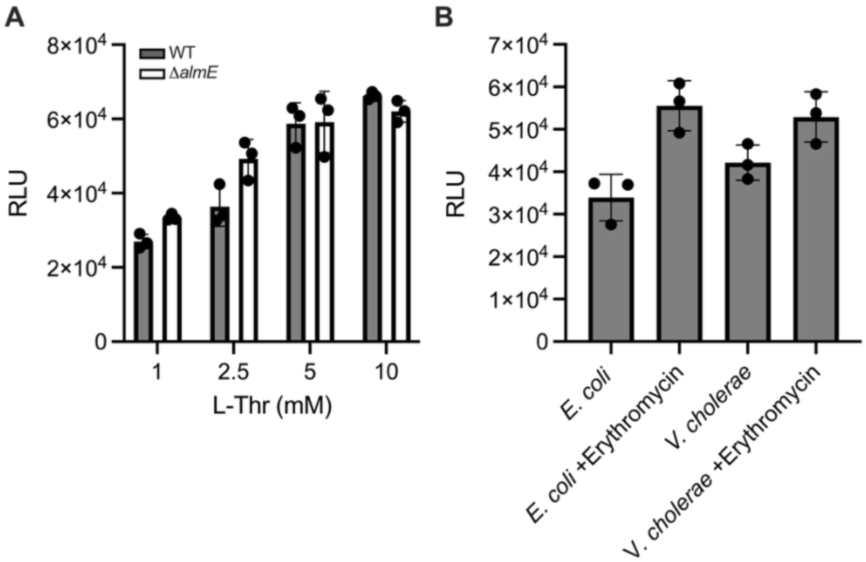

Figure S7: WT and Δ*almE V. cholerae* produce the same amount of DPO activity and administration of erythromycin increases DPO activity. (A) Lux bioassay quantitation of activity in cell-free culture fluids of WT and Δ*almE V. cholerae* supplemented with the indicated concentrations of L-Thr. (B) Quantitation of Lux bioassay activity in cell-free culture fluids of *V. cholerae* and *E. coli* strains supplemented with 2.5 mM L-Thr and grown with or without 2 µg mL^{-1} and 8 µg mL^{-1} of erythromycin, respectively. RLU denotes relative light units. In both panels, error bars denote standard deviations of three biological replicates.

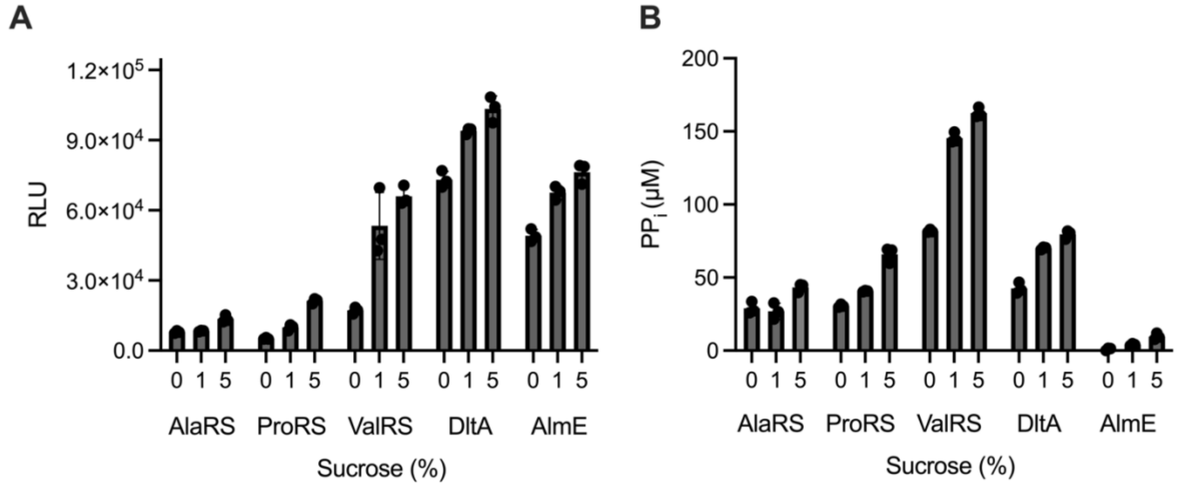

Figure S8: A crowding agent increases the catalytic and spontaneous steps required for DPO production. *In vitro* reactions were carried out as in Figure 2 with the AlaRS, ProRS, ValRS, DltA, and AlmE enzymes in the presence of 0-5% (*w/v*) sucrose. (A) Quantitation of activity in the Lux bioassay at 2 h. RLU denotes relative light units. (B) Quantitation of PP_i production as assessed by the malachite green assay at 2 h. [PP_i] is a proxy for [$^{13}C_3$-Ala-AMP] (see legend to Figure 2B). See Figure S1B for P_i standard curves. All reactions were normalized to samples lacking enzyme. In both panels, error bars denote standard deviations of three technical replicates.

Table S1: Plasmid list

Plasmid stock name	Plasmid name	Relevant fragment	Comment	Origin, marker	Ribosome binding site	References
Ec-DLS_100	pET15b-pT7-Ec-6XHIS::*alaS*	6XHIS::*alaS*	AlaRS expression plasmid	pBR322, Amp^R	T7	2
Ec-DLS_083	pET15b-pT7-Ec-6XHIS::368 *alaS*	6XHIS::368 *alaS*	AlaRS 368N expression plasmid	pBR322, Amp^R	T7	3
Ec-DLS_129	pET-28b(+)-pt7-6XHIS::*ddlB*	6XHIS::*ddlB*	DdlB expression plasmid	pBR322, Kan^R	T7	Genescript
Ec-DLS_88	pET15b-pT7-*proS*::6XHIS	*proS*::6XHIS	ProRS expression plasmid	pBR322, Amp^R	T7	2
Ec-DLS_93	pSC101-pT7-*serS*::6XHIS	*serS*::6XHIS	SerRS expression plasmid	pSC101, Amp^R	T7	2
Ec-DLS_114	pSC101-pT7-*valS*::6XHIS	*valS*::6XHIS	ValRS expression plasmid	pSC101, Amp^R	T7	2
Ec-DLS_107	pET15b-pT7-*tyrS*::6XHIS	*tyrS*::6XHIS	TyrRS expression plasmid	pBR322, Amp^R	T7	2
Ec-DLS_127	pQE-60-pT5-*dltA*::6XHIS	*dltA*::6XHIS	DltA expression plasmid	ColE1, Amp^R	T5	Genescript
Ec-DLS_148	pET28b(+)-pT7-6XHIS::*almE*	6XHIS::*almE*	AlmE expression plasmid	pBR322, Kan^R	T7	Genescript

Table S2: Strain list

Strain	Relevant markers / genotype	Reference / source
V. cholerae		
Vc-DLS_003	Δ*vqmA* Δ*tdh lacZ*::P*vqmR-lux* *1807*::pBAD-*vqmA*	4
Vc-DLS_001	Wild-type C6706	5
Vc-DLS_010	Δ*almE*	6
E. coli		
Ec-DLS_135	Wild-type BW25113	7
BL21 (DE3)	*E. coli* B F^- *ompT* *hsdS*(r_B^- m_B^-) dcm^+ Tet^r *gal* λ(DE3) *endA* Hte	Aligent

Table S3: Primer and oligonucleotide list

Name	Sequence	Description
WT_tRNA^{Ala}	GAAATTAATACGACTCACTATAGGGGCTATAGCTCAGCTGGGAGAGCGCCTGCTTTGCACGCAGGAGGTCTGCGGTTCGATCCCGCATAGCTCCACCA	tRNA^{Ala} dsDNA oligonucleotide (IDT)
Mutant_ tRNA$^{AlaU3:G70}$	GAAATTAATACGACTCACTATAGGTGCTATAGCTCAGCTGGGAGAGCGCCTGCTTTGCACGCAGGAGGTCTGCGGTTCGATCCCGCATAGCGCCACCA	tRNA$^{AlaU3:G70}$ dsDNA oligonucleotide (IDT)
AlaRS368N_forward	TAACAAAGCCCGAAAGGAAGC	AlaRS 368N plasmid construction
AlaRS368N_reverse	AGCAAACTGCTCTTCTTCAGTCTT	AlaRS 368N plasmid construction

References

1. Neuhaus, F. C.; Lynch, J. L. THE ENZYMATIC SYNTHESIS OF D-ALANYL-D-ALANINE. 3. ON THE INHIBITION OF D-ALANYL-D-ALANINE SYNTHETASE BY THE ANTIBIOTIC D-CYCLOSERINE. *Biochemistry* **1964**, *3*, 471–480. https://doi.org/10.1021/bi00892a001.
2. Villarreal, F.; Contreras-Llano, L. E.; Chavez, M.; Ding, Y.; Fan, J.; Pan, T.; Tan, C. Synthetic Microbial Consortia Enable Rapid Assembly of Pure Translation Machinery. *Nat Chem Biol* **2018**, *14* (1), 29–35. https://doi.org/10.1038/nchembio.2514.
3. Regan, L.; Bowie, J.; Schimmel, P. Polypeptide Sequences Essential for RNA Recognition by an Enzyme. *Science* **1987**, *235* (4796), 1651–1653. https://doi.org/10.1126/science.2435005.
4. Mashruwala, A. A.; Decker, K.; Fei, C.; Valastayan, J.; Bassler, B. L. A Transcription Factor-sRNA-Mediated Double-Negative Feedback Loop Confers Pathogen-Specific Control of Quorum-Sensing Genes. *bioRxiv* **2025**, 2025.08.22.671807. https://doi.org/10.1101/2025.08.22.671807.
5. Thelin, K. H.; Taylor, R. K. Toxin-Coregulated Pilus, but Not Mannose-Sensitive Hemagglutinin, Is Required for Colonization by Vibrio Cholerae O1 El Tor Biotype and O139 Strains. *Infect Immun* **1996**, *64* (7), 2853–2856. https://doi.org/10.1128/iai.64.7.2853-2856.1996.
6. Grant, N. A.; Donkor, G. Y.; Sontz, J. T.; Soto, W.; Waters, C. M. Deployment of a Vibrio Cholerae Ordered Transposon Mutant Library in a Quorum-Competent Genetic Background. *bioRxiv* **2024**, 2023.10.31.564941. https://doi.org/10.1101/2023.10.31.564941.
7. Datsenko, K. A.; Wanner, B. L. One-Step Inactivation of Chromosomal Genes in Escherichia Coli K-12 Using PCR Products. *Proceedings of the National Academy of Sciences* **2000**, *97* (12), 6640–6645. https://doi.org/10.1073/pnas.120163297.
